# Urbanization of Scrub Typhus Disease in South Korea

**DOI:** 10.1371/journal.pntd.0003814

**Published:** 2015-05-22

**Authors:** Sang-Won Park, Na-Young Ha, Boyeong Ryu, Ji Hwan Bang, Hoyeon Song, Yuri Kim, Gwanghun Kim, Myoung-don Oh, Nam-Hyuk Cho, Jong-koo Lee

**Affiliations:** 1 Department of Internal Medicine, Seoul National University College of Medicine, Seoul, Republic of Korea; 2 Department of Internal Medicine, Boramae Medical Center, Seoul, Republic of Korea; 3 Department of Microbiology and Immunology, Seoul National University College of Medicine, Seoul, Republic of Korea; 4 Department of Biomedical Science, Seoul National University College of Medicine and Bundang Hospital, Seoul, Republic of Korea; 5 Medical Research Center, Seoul National University College of Medicine, Seoul, Republic of Korea; 6 Seoul Center for Infectious Disease Control, Seoul, Republic of Korea; 7 Department of Family Medicine, Seoul National University College of Medicine, Seoul, Republic of Korea; Australian National University, AUSTRALIA

## Abstract

**Background:**

Scrub typhus is an endemic disease in Asia. It has been a rural disease, but indigenous urban cases have been observed in Seoul, South Korea. Urban scrub typhus may have a significant impact because of the large population.

**Methods:**

Indigenous urban scrub typhus was epidemiologically identified in Seoul, the largest metropolitan city in South Korea, using national notifiable disease data from 2010 to 2013. For detailed analysis of clinical features, patients from one hospital that reported the majority of cases were selected and compared to a historic control group. Chigger mites were prospectively collected in the city using a direct chigger mite-collecting trap, and identified using both phenotypic and 18S rDNA sequencing analyses. Their infection with *Orientia tsutsugamushi* was confirmed by sequencing the 56-kDa antigen gene.

**Results:**

Eighty-eight cases of urban scrub typhus were determined in Seoul. The possible sites of infection were mountainous areas (56.8%), city parks (20.5%), the vicinity of one’s own residence (17.0%), and riversides (5.7%). Eighty-seven chigger mites were collected in Gwanak mountain, one of the suspected infection sites in southern Seoul, and seventy-six (87.4%) of them were identified as *Helenicula miyagawai* and eight (9.2%) as *Leptotrombidium scutellare*. Pooled DNA extracted from *H*. *miyagawai* mites yielded *O*. *tsutsugamushi* Boryong strain. Twenty-six patients from one hospital showed low APACHE II score (3.4 ± 2.7), low complication rate (3.8%), and no hypokalemia.

**Conclusions:**

We identified the presence of indigenous urban scrub typhus in Seoul, and a subgroup of them had mild clinical features. The chigger mite *H*. *miyagawai* infected with *O*. *tsutsugamushi* within the city was found. In endemic area, urban scrub typhus needs to be considered as one of the differential febrile diseases and a target for prevention.

## Author Summary

Scrub typhus is one of the high burdened infectious diseases in endemic rural areas. Our study showed that the disease could be endemic in urban areas. Indigenous urban scrub typhus may also have significant impact in terms of a disease burden and differential diagnosis. A low rate of characteristic eschar and skin rash may make the clinical suspicion more troublesome. Scrub typhus is the 3^rd^ most frequent notifiable infectious disease in South Korea. Seoul is a densely populated capital city located in northwestern South Korea. We epidemiologically identified indigenous urban cases. A subgroup of them showed mild clinical characteristics. Direct mite collection from a suspected infection site, Mt. Gwanak in Seoul, showed that *Helenicula miyagawai* (87.4%) and *Leptotrombidium scutellare* (9.2%) were the dominant species. In addition, *O*. *tsutsugamushi*-specific gene, *tsa56*, was identified in a fraction of the collected *H*. *miyagawai*. The 18S rDNA sequencing of the chigger mites was complementarily helpful for the differentiation of species. Our data are from one region, but urban scrub typhus is likely a scenario in endemic areas. Urban areas should be included as possible locations for scrub typhus.

## Introduction

Scrub typhus, also known as tsutsugamushi disease, is a mite-borne infectious disease that is endemic in a triangular geographic region containing the Indian subcontinent, northern Australia and the Far East. In endemic areas, the risk of infection is mainly associated with farming and outdoor activities in rural areas, which have a high disease burden [1–4]. In non-endemic area, scrub typhus is one of the most important febrile diseases affecting returning travelers [5]. Clinical clues that lead to a diagnosis include maculopapular skin rash, regional lymphadenopathy, and eschar. The presence of characteristic eschar varies according to the geographical region [6–9]. Low eschar positivity makes proper diagnostic suspicion difficult. The endemicity of scrub typhus is closely associated with the habitation of vector mites carrying the causative agent, *Orientia tsutsugamushi*. The major vector mites (the *Leptotrombidium* species) can often be collected from the mountainous regions and rural farm lands within endemic regions. If a disease flows into a densely populated urban area and attains its endemicity, it will have a significant impact in terms of a disease burden and differential diagnosis in clinical practices. Recently, urban scrub typhus cases that were suspected to occur within Seoul, the largest city in South Korea, have been frequently noted in clinical practices. Seoul is the capital city of South Korea which is located in the northwestern region. It is a densely populated metropolitan city with a population of 10.369 million people (in 2014) living in a land area of 605.21 km^2^ (http://stat.seoul.go.kr/jsp3/index.jsp), which is comparable in density to Manhattan in the United States with a population of 1.585 million people (in 2010) in an area of 59.5 km^2^. In South Korea, scrub typhus was first reported in 1951 during the Korean War [10], and it has re-emerged since 1986 [11]. The incidence of scrub typhus has increased steadily [12], and it was the 3^rd^ most frequent notifiable infectious disease in South Korea in 2012 (S1 Table) [13]. There have been studies about urban scrub typhus in the literature [14–16]. Our study was conducted to investigate the status of indigenous urban scrub typhus and its clinical characteristics in Seoul at more northern latitude. In addition, we prospectively determined the causative vector mites within the city.

## Methods

### Epidemiologic investigation

Confirmed and clinical cases of scrub typhus reported to and investigated by the local health bureau and the Korea Centers for Disease Control and Prevention (KCDC) from 2010 to 2013 in Seoul been analyzed retrospectively. An epidemiologic investigation was conducted by public health officers in district public health centers using a questionnaire with details of outdoor exposure histories. The maximal incubation period for the possible exposure was set at one month. Only the cases suspected to be acquired within the metropolitan city of Seoul were identified. The putative site of infection was estimated in view of scrub or vegetation area which was in the scope of patient’s activity. The case having multiple suspected sites of both within and outside Seoul was not considered to be of urban origin. The site of infection was marked in a map overlapped with biotope levels (http://urban.seoul.go.kr/4DUPIS/sub7/sub7_7_1.jsp) and summarized in S2 Table. The geographic location of infection was pointed using Google Earth (http://earth.google.com), and the map was drawn using QGIS (version 2.2, http://qgis.org) and PowerPoint (2013, Microsoft). The biotope level is determined according to the land assessment by a combined analysis of urban ecological characteristics and indicates the grade of value in terms of nature conservation. The range of biotope level is determined differently depending on a policy of a city [17]. In Seoul, we used a city map having biotope levels ranging from 1 to 3. Level-1 indicates a natural area to be protected from any land exploitation, level-2 is a partially-exploited area to be conserved or restored, and level-3 indicates an exploited or artificially landscaped area including agricultural farms and city parks (http://urban.seoul.go.kr/4DUPIS/sub7/sub7_7_1.jsp). The district map of Seoul was obtained from public SGIS services of Statistics Korea (http://sgis.kostat.go.kr). To estimate the influence of scrub typhus incidence in neighboring regions on Seoul, the temporal change of national and regional incidence of scrub typhus per 100,000 individuals of the general population from 2001 to 2012 was calculated using the National Notifiable Disease Surveillance System (NNDSS) data (http://is.cdc.go.kr) and the national census (http://kosis.kr).

### Clinical characteristics of urban scrub typhus

To compare the clinical characteristics of urban vs. rural scrub typhus, clinical data were collected from patients cared for from 2010 to 2012 in one university-affiliated hospital, Boramae Medical Center, located in the southern part of Seoul. It reported a majority of scrub typhus patients determined to be urban infection. The characteristics included age, sex, underlying diseases, the location of eschar, treatment, laboratory findings, the severity of the disease, complications, and clinical outcome. The data were collected retrospectively for this study, but the patients were assessed using a fixed questionnaire format in routine clinical practices. For the comparison of patient characteristics, we used our previous control group. The control group included only eschar-positive scrub typhus patients confirmed by eschar-based polymerase chain reaction (PCR). The data were collected prospectively in 2006 and were mostly from rural endemic areas outside Seoul [18]. The participating hospitals for the control group were Sanggye Paik Hospital, Ilsan Paik Hospital, Pusan Paik Hospital, Dongguk University Ilsan Hospital, Dankook University Hospital, Namwon Medical Center, Chonbuk National University Hospital, and Sunlin Hospital [18].

### Definition

Clinical scrub typhus was defined as a case with typical eschar and at least two of the following manifestations: fever, maculopapular skin rash, regional lymphadenopathy, headache, myalgia, cough, and abdominal discomfort. Acute therapeutic response to doxycycline or azithromycin with no other alternative diagnosis was a basic component of the clinical scrub typhus. Confirmed case was defined as positive serological result for *O*. *tsutsugamushi*. The positive serology was as a four-fold or greater change in the titer of paired sera, or a single cut-off titer of IgM antibody ≥ 1:160 from an indirect immunofluorescence antibody assay against a mixture of *O*. *tsutsugamushi* antigens (Gilliam, Karp, Kato and Boryong) in clinical scrub typhus patients [19]. The clinical severity was assessed by the Acute Physiology and Chronic Health Evaluation (APACHE) II score which was measured within 24 hours of initial admission [20]. Complication was defined as a new onset of problems and conditions as below. The central nervous system (CNS) complication was defined as the presence of altered mental state, seizure, intracranial hemorrhage and/or infarct. Respiratory complication was defined as the presence of lung infiltration and at least one of the followings: arterial PaO_2_/FiO_2_ ≤ 250, respiratory rate ≥ 30/min, or requirement for mechanical ventilation. Cardiac complication was defined as any presence of myocarditis, arrhythmia, or ischemic heart diseases. Myocarditis was suspected if there was any abnormality of electrocardiography (ECG), serum levels of troponin or creatine phosphokinase (CK) fractions and an echocardiogram. The criteria for possible acute myocarditis was one or more of the following: elevation of troponin or CK-MB, ECG findings suggestive of acute injury, and reduced left ventricular ejection fraction or regional wall motion on cardiac imaging. Probable acute myocarditis was diagnosed when the above criteria were met and accompanied also by cardiac symptoms, such as shortness of breath or chest pain. Endomyocardial biopsy for definite myocarditis was rarely considered. Renal complication was defined as serum creatinine level ≥ 2.0 mg/dL or the aggravation of pre-existing renal injury resulting in acute renal replacement therapy. Septic shock was defined as the need for parenteral vasopressor to maintain systolic blood pressure above 90 mm of Hg for more than 1 hour. Underlying diseases with known or newly discovered below specific conditions were included: diabetes mellitus (DM) requiring insulin or oral glucose lowering agents therapy; hypertension (HTN) requiring antihypertensive medication; cerebrovascular diseases (CVD) with residual complications; symptomatic congestive heart failure (CHF) requiring medication; chronic liver diseases (CLD) including liver cirrhosis and chronic active hepatitis; bronchial asthma and chronic obstructive lung disease (COPD) requiring maintenance medication [18].

### Collection and identification of chigger mites

Chigger mites were collected to ensure their presence and identify their infection with *O*. *tsutsugamushi* within Seoul using a chigger mite collecting trap which includes BG Lure (BioGents GmbH, Regensburs, Germany) mimicking human skin odor and adhesive tape on the surface. The location of traps in the Gwanak mountain was selected based on the most frequent areas for infected cases in 2012 after personal interviews with the reported patients. A total of fifteen traps were set up at three points, with five traps each in the northern foot of Mt. Gwanak for two months from October to December 2013. Mt. Gwanak is located on the southern border of Seoul and is 632 meters high. Adhesive tape on the surface of mite trap was collected weekly. The chigger mites were removed from the tape under a microscope, placed in 80% ethanol, mounted on glass slides with mounting media, and then identified to species level at 400× magnification using the morphological key features for chigger mites in Korea [21]. Autofluorescent images of chigger mites were obtained using a confocal laser scanning microscope (Olympus FV1000, Olympus, Japan) at excitation wavelength of 488 nm. A wide emission wavelength ranging between 500 and 590 nm was used to collect as much fluorescence as possible [22]. Images were analyzed using Fluoview software (Olympus, Japan). For the genetic identification of individual chigger mites, total DNA was extracted from each mite using a slight modification of a previously described method [23]. Briefly, the individual chigger mite was punctured with a fine needle under a stereomicroscope and digested with proteinase K (2 mg/ml) in lysis buffer (100 mM Tris—Cl, pH 8.0, 160 mM sucrose, 80 mM EDTA, and 0.5% SDS) for 18 h at 56°C. The total DNA was extracted from each chigger mite using a QIAamp DNA Mini Kit (QIAGEN, Hilden, Germany) according to the manufacturer’s protocol. Universal primers for amplifying arthropod 18S rDNA were used (forward: CTGGTTGATYCTGCCAGT; reverse: TCTCAGGCTCCYTCTCCGG; 400~450 bp) [24].

### Identification of *O*. *tsutsugamushi* in mites

For the genetic identification of *O*. *tsutsugamushi* from individual chigger mites, the total DNA was extracted from each mite. DNA sample from each mite or pooled DNA (up to ten mites) from the same mite species was used to detect the 56-kDa antigen gene (*tsa56*) of *O*. *tsutsugamushi* by PCR analysis (S3 Table). Primers for detecting *tsa56* had previously been constructed in our laboratory based on the common conserved region of the prototype strains of *O*. *tsutsugamushi* and were used as follows; *tsa56* (forward: ATGAAAAAAATTATGTTAATT; reverse: CAATTTAACAAGATCTTTAT; 1164 bp). The analysis of the derived nucleotide sequences was performed for matching genotypes using the NCBI-BLAST service (http://blast.ncbi.nlm.nih.gov/Blast.cgi).

### Statistical analysis

Univariate comparison was performed using the χ^2^ test or Fisher’s exact test for categorical variables. Continuous variables were compared using Student’s t-test. All statistical tests were two-tailed, and P values < 0.05 were considered significant. The data were analyzed using SPSS (version 20; IBM Corporation).

### Ethics statement

This study was approved by the institutional review boards (IRB) of Boramae Medical Center (20140929/26-2014-117/102) and Seoul National University Hospital (E-1406-067-588). Consent from the study subjects was exempted by both IRBs because the data were collected retrospectively, all subjects couldn’t be contacted at the time of data collection, the data didn’t include sensitive personal information, and all personal identifiers were anonymized for confidentiality before the data process.

## Results

### Epidemiologic characteristics

**Fig 1:**
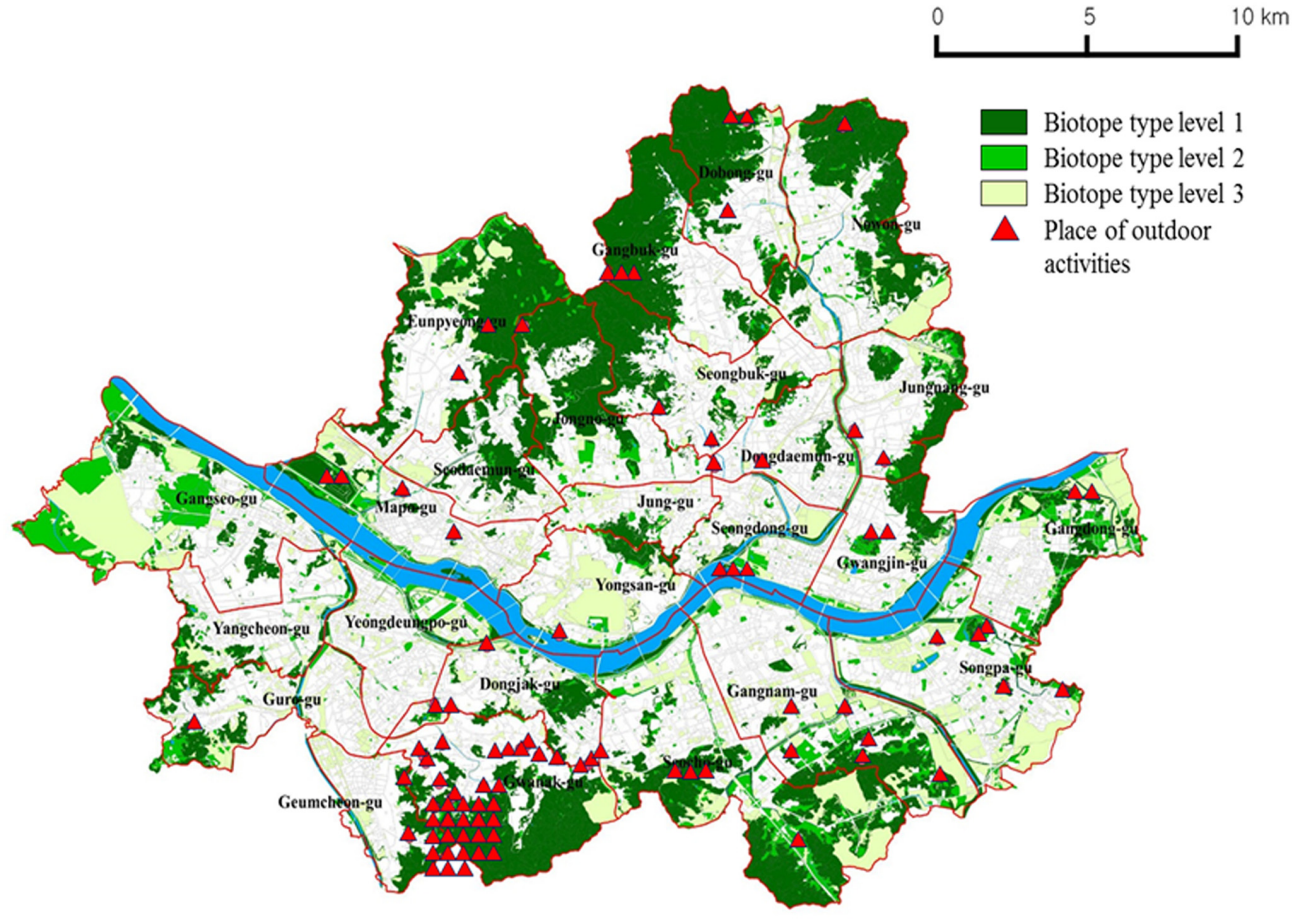
The spatial distribution of scrub typhus cases with the epidemiological linkage to outdoor activities in Seoul and biotope levels during 2010–2013. Infection mostly occurred in the biotope level-1 area, which is a suitable place for mite survival.

From 2010 to 2013, a total of 29,791 cases of scrub typhus throughout the nation were reported to the KCDC. In Seoul, 1,110 cases were reported during the same period. Among them, eighty-eight (7.9%) cases were estimated to be acquired within Seoul based on the maximal incubation period and the type and extent of individual exposure history. Fifty-three patients were confirmed serologically by seroconversion of paired sample or positive single IgM titer, and thirty-five were clinical scrub typhus (S2 Table). The number of patients from 2010 to 2013 was sixteen, ten, thirty-three and twenty-nine, respectively. Of the twenty-five districts within Seoul, eighteen districts had at least one case of urban scrub typhus during the four year period. Among the eighty-eight cases, the estimated sites of infection within the city were mountainous areas (fifty cases, 56.8%), city parks (eighteen cases, 20.5%), the vicinity of one’s own residence (fifteen cases, 17.0%), and riversides (five cases, 5.7%). As depicted in Fig 1, these sites are mostly located in the biotope level-1 area. The occupations in the order of frequency were housewife (thirty-six, 40.9%), none (sixteen, 18.2%), office worker (six, 6.8%), private business (six, 6.8%), construction worker (six, 6.8%) and others of minority (S2 Table).

Meanwhile regarding nationwide data, the national incidence of scrub typhus per 10^5^ individuals was 5.7 cases in 2001, 13.7 cases in 2006, and 17.7 cases in 2012. In southwestern rice and dry field areas, the incidence per 10^5^ in provinces or metropolitan cities were 15.7 to 20.1 cases in 2001 and 36.7 to 72.9 cases in 2012. In northeastern mountainous areas (Kangwon Province), the incidence per 10^5^ remained within 3~4 cases during the interval period. In the higher latitudinal western area (Gyeonggi Province) surrounding the metropolitan city of Seoul, the incidence per 10^5^ was 1.5 cases in 2001 and 9.1 cases in 2012.

### Clinical characteristics of urban scrub typhus

Among the eighty-eight patients, twenty-six patients were reported from Boramae Medical Center during 2010 to 2012. Among twenty-six patients, twelve had seroconversion of paired sample, four had positive single IgM titer, and ten were clinical cases. All patients presented with fever. Eschar was found in twenty-three (88.5%) patients. Skin rash was observed in twenty-five (96.2%) patients. Regional lymphadenopathy was noted in only three (11.5%) patients. Based on the laboratory results, hypokalemia or acute kidney injury was not present (Table 1). All patients responded well to antibiotic therapy, and there was no case fatality. One patient took azithromycin, but the other patients took doxycycline of seven days. Compared with control group, regional lymphadenopathy (11.5%), hypokalemia (0%), APACHE II score (3.4 ± 2.7) and accompanying complications (3.8%) were significantly lower in the study group in a univariate analysis (Table 1). The only complication was one case of upper gastrointestinal bleeding. The control group had sixteen cases of respiratory (7.8%), CNS (5.9%), cardiac (2.0%), and septic shock (1.3%) complications. The twenty-six patients of urban scrub typhus were grouped into two groups, confirmed (n = 16) and clinical (n = 10) cases, to compare the difference regarding the same clinical variables. There was no significant difference between two groups (Table 1). The possible risk behavior that lead to predisposition of the infection included walking in the mountainous area (eleven cases; 42.3%), gardening (seven cases; 26.9%), playing in the park (four cases; 15.4%), and trivial harvesting (four cases; 15.4%).

**Table 1:** Clinical characteristics of urban scrub typhus compared with control patients.

Variable		Study patients			Control patients	
		Group-1, n = 10	Group-2, n = 16	Total, n = 26	n = 153	*P* ^b^
Age, years^a^		49 (39–66)	58 (45–69)	56 (44–68)	63 (49–71)	0.114
Sex, male		4 (40.0)	8 (50.0)	12 (46.2)	63 (41.2)	0.634
Underlying diseases		3 (30.0)	7 (43.8)	10 (38.5)	55 (35.9)	0.805
Diabetes mellitus		2 (20.0)	2 (12.5)	4 (15.4)	13 (8.5)	0.279
Hypertension		2 (20.0)	5 (31.3)	7 (26.9)	33 (21.6)	0.545
Others^c^		0 (0)	3 (18.8)	3 (11.5)	23 (15.0)	0.772
Type of visit, ER or admission		10 (100)	12 (75.0)	22 (84.6)	137 (89.5)	0.499
Duration of hospital stay, days^a^		1.4 (0–3.3)	3.4 (0.8–5.5)	2.5 (0–4.0)	6.7 (3.0–7.0)	0.154
Time from initial Sx^d^ to visit, days^a^		7.4 (4.5–10.3)	8.3 (6.0–10.0)	7.9 (5.0–10.0)	6.9 (4.0–9.0)	0.258
Eschar		10 (100)	13 (81.3)	23 (88.5)	153 (100)	-
Location, trunk		6 (60.0)	4 (30.8)	10 (38.5)	94 (61.4)	0.087
Fever		10 (100)	16 (100)	26 (100)	153 (100)	-
Skin rash, maculopapular		10 (100)	15 (93.8)	25 (96.2)	139 (90.8)	0.700
Regional lymphadenopathy		1 (10.0)	2 (12.5)	3 (11.5)	50 (32.7)	0.035
Laboratory values						
	WBC, >10 K or <4 K/mm^3^	3 (30.0)	2 (12.5)	5 (19.2))	57 (37.3)	0.080
	Hemoglobin ≤10 g/dL	0 (0)	0 (0)	0 (0)	8 (5.2)	0.605
	Platelet ≤100 K/mm^3^	0 (0)	3 (18.8)	3 (11.5)	37 (24.2)	0.205
	AST >40 IU/L	8 (80.0)	11 (68.3)	19 (73.1)	128 (83.7)	0.193
	ALT >40 IU/L	5 (50.0)	10 (62.5)	15 (57.7)	114 (74.5)	0.077
	CRP >10 mg/dL	1 (10.0)	7 (43.8)	8 (30.8)	39 (27.9)	0.762
	Potassium ≤3.5 mmol/L	0 (0)	0 (0)	0 (0)	35 (22.9)	0.009
	Serum creatinine >2.0 mg/dL	0 (0)	0 (0)	0 (0)	3 (2.0)	-
APACHE II score^a^		2.5 (0–5.0)	4.0 (1.3–6.0)	3.4 (1.0–5.3)	6.6 (4.0–9.0)	<0.001
Complications		0 (0)	1 (6.3)	1 (3.8)	34 (22.2)	0.031
Fatality		0 (0)	0 (0)	0 (0)	0 (0)	-

^a^The function is mean (IQR).

^b^ *p*-value: comparison between n = 26 and n = 153.

^c^'Others' indicates a group of less frequent chronic diseases including congestive heart failure, cerebrovascular disease, chronic liver disease, asthma, and chronic obstructive pulmonary disease.

^d^'Sx' indicates any type of scrub typhus-related clinical manifestation.

IQR = interquartile range; ER = emergency room; SD = standard deviation; WBC = white blood cell; AST = aspartate aminotransferase; ALT = alanine aminotransferase; CRP = C-reactive protein

### Collection of chigger mites and identification of *O*. *tsutsugamushi*

**Fig 2:**
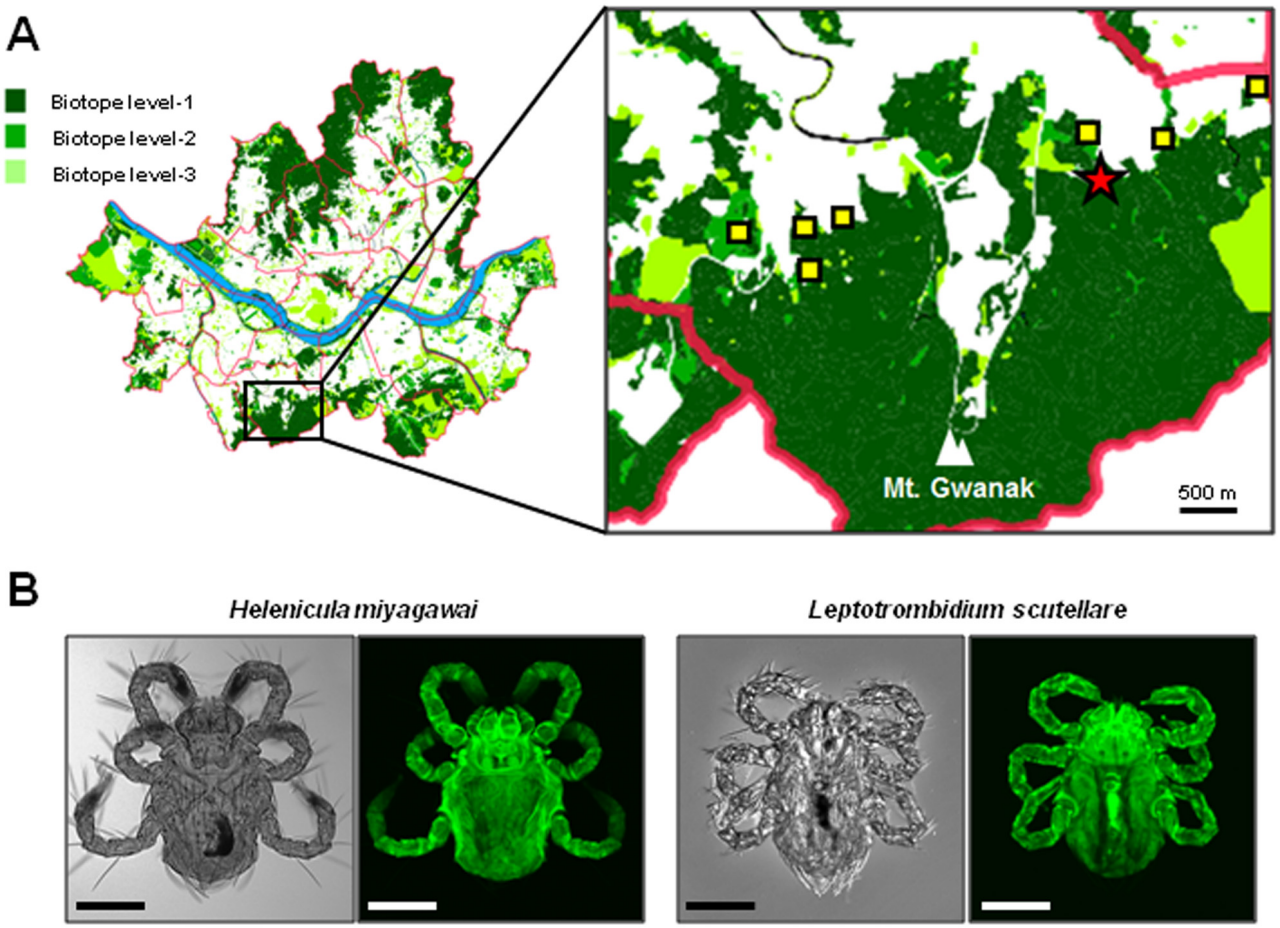
Collection of chigger mites in Mt. Gwanak. (A) Map of scrub typhus infection in the metropolitan city of Seoul from 2010 to 2013. Among 1,110 patients interviewed, 88 cases were suspected to occur during outdoor activity within Seoul. Eight potential sites of infection (quadrangle and star) identified in 2012 from nine cases and the location of chigger mite collection (star) on the foot of Mt. Gwanak are presented in the right panel. The district map of Seoul was obtained from public SGIS services of Statistics Korea (http://sgis.kostat.go.kr). (B) Representative microscopic images of *Helenicula miyagawai* and *Leptotrombidium scutellare* collected in this study. Differential interference contrast (DIC) images (left panels) and autofluorescent images (right panels) of the chigger mite were taken using light microscopy and confocal laser scanning microscopy, respectively. Bar: 100 μm.

Nine cases of scrub typhus from eight sites at the foot of Mt. Gwanak were identified in 2012, and chigger mites were collected from one of these nine sites (Fig 2A). Approximately 100 chigger mites were initially collected from the traps, but only eighty-seven mites were subjected to final species analysis due to individual damage during handling. Among the eighty-seven mites, seventy-six (87.4%) were identified as *Helenicula miyagawai*, eight (9.2%) as *L*. *scutellare*, two (2.3%) as *L*. *zetum*, and one (1.1%) as *L*. *palpale* (Fig 2B). Among seventy-six *H*. *miyagawai* mites, thirty-eight (50%) were confirmed by the genotypic PCR method based on 18S rDNA sequences and their nucleotide sequences were all identical. Seven (87.5%) of the eight *L*. *scutellare* were genotypically confirmed, and they showed all the same nucleotide sequences. Two mites of *L*. *zetum* and one mite of *L*. *palpale* were only morphologically identified. PCR analysis for the *tsa56* gene of *O*. *tsutsugamushi* using the extract of individual mites failed to detect any amplification product. However, two batches of a pooled DNA from ten *H*. *miyagawai* showed positive products (S3 Table). The two nucleotide sequences were identical and belonged to Boryong strain. When the sequence was compared with a prototype Boryong sequence (NCBI accession number AM494475), there were differences at four nucleotides. The new sequence was named ‘*O*. *tsutsugamushi* genotype Gwanak’ after the place of its first isolation. The nucleotide sequences established in this study were deposited in GenBank under accession numbers of KM111301 (*L*. *scutellare*), KM111302 (*H*. *miyagawai*), and KM111303 (*O*. *tsutsugamushi* Gwanak).

## Discussion

Scrub typhus is caused by *O*. *tsutsugamushi*, which is transmitted by the trombiculid chigger mite during its human bite. *O*. *tsutsugamushi* is naturally maintained within the life cycle of mites [25]. It can be transovarially transmitted in mites. The incidence of scrub typhus is influenced by multiple factors. The behavior and population densities of Trombiculidae and wild rodents and the types of human activity may affect the infection. There have been surveys investigating the distribution of wild rodents and Trombiculidae in South Korea [23,26]. *Apodemus agrarius* is a dominant wild rodent harboring chigger mites with *O*. *tsutsugamushi*. Its typical habitats are forests, cornfields and other agricultural land, but urban areas are also well-adapted sites [27]. Trombiculidae have a nationwide distribution, and seven species (*L*. *pallidum*, *L*. *scutellare*, *L*. *palpale*, *L*. *orientale*, *L*. *zetum*, *Eushoengastia koreaensis*, and *Neotrombicula japonica*) can carry *O*. *tsutsugamushi* in South Korea [28]. The dominant species of Trombiculidae primarily responsible for *O*. *tsutsugamushi* infection in South Korea are *L*. *pallidum* in the colder central area and *L*. *scutellare* in the warmer southern area. Dry fields and the surrounding bush and grassy land have been identified as risk areas for the high frequency of *O*. *tsutsugamushi* infection [12,29]. These environments seem to be appropriate places for the survival of the vectors. As described in the results section in this study, rural areas in southern and southwestern fields of South Korea have a high incidence of scrub typhus, and recent trends indicate that the high incidence areas are expanding. The trend of expansion includes a higher density of pre-existing endemic areas and an upward expansion to the northern region. This trend was also shown recently by Jin et al using geographic information system (GIS)-based spatial analysis [30]. Global warming might have contributed to this expansion. An annual average temperate of 10°C was estimated to be a northern limit for the distribution of the warm temperature adapted *L*. *scutellare*, and an overall increase in temperature may promote the expansion of its habitat to the north [28]. The urban environment is different from rural areas. However, the eco-friendly trend of having more natural parks within the city and preserving the natural environment surrounding the city may create more suitable habitats for vectors and small rodents [31–33]. Seoul has such geographical characteristics as a river (Han River) passing through the city and relatively large peaks surrounding the city accompanied by dry fields at the peak’s base. Under the high infection pressure of surrounding suburbs, the inflow of the vectors into the city may only be a matter of time. Current presence of urban scrub typhus could be interpreted as an incursion of vectors into the urban setting or an incursion of an expanding human population into relatively less habitable natural areas. As described in the introduction section, Seoul is a densely populated area and there has been no room for further expansion into less inhabitable natural area within Seoul. The area around Mt. Gwanak is one of the populated residential area since long ago. The estimated sites of infection depicted in the Fig 1 showed their central locations within the city. There has been little change in the aspect of human contacts but the urban scrub typhus is a new phenomenon. Seroprevalence study in the urban area deserves consideration [34,35]. Although it can’t differentiate urban vs. non-urban infection under free moving society, the seroprevalence study combined with serial monitoring of reported patients will help estimate the size of urban exposure to scrub typhus. The comparison of intra-municipal locations may indicate risk area.

Lee et al surveyed the distribution of chigger mites infesting rodents in Mt. Gwanak (previously named Kwan-ak) in southern Seoul in 1986 and 1987 [36]. The order of frequency was *L*. *orientale* (31.4%), *L*. *palpale* (30.0%), *L*. *zetum* (5.41%), *L*. *scutellare* (0.34%), and *H*. *miyagawai* (0.13%). The dominant rat was *A*. *agrarius* (93.6%). Because we collected mites directly from the field instead of rodents, direct comparison of their data with ours is not exact. However, as the mites were collected in the same area and the area was the most common site of scrub typhus infection in our study population (depicted in Fig 1), the spot proportion of chigger mites collected may provide indirect insight for the infectious vectors. In this context, the 2^nd^ highest proportion of *L*. *scutellare* that is warmer area type is noteworthy. The high proportion of *H*. *miyagawai* (87.4%) is also interesting. Our study showed for the first time that *H*. *miyagawai* contained *O*. *tsutsugamushi* (genotype Boryong) and that chigger mites infected with *O*. *tsutsugamushi* were present within Seoul. The PCR results using individual mites were all negative, but two batches of pooled mites from *H*. *miyagawai* were positive for *O*. *tsutsugamushi*. Considering that the positive rates for *O*. *tsutsugamushi* in chigger mites from major endemic areas were 0.9~1.5% in previous studies [37,38], the rate of infected chigger mites in high-risk areas in Seoul is comparable to that of rural areas. This means that the average rate of urban infection is low but the risk of infection in certain areas is high. These “certain areas” may include mountainous areas, city parks, and riversides. *H*. *miyagawai* characteristically forms larval clusters on the lower parts of vegetation, which allows easy boarding onto host animals. It then infests wild mammals, birds, and domesticated cats and dogs [39]. The high proportion of *H*. *miyagawai* in the actual high incidence area of scrub typhus combined with the carriage of *O*. *tsutsugamushi*, especially the dominant Boryong genotype, suggests that this mite may play an actual role in human infection within Seoul. However, this possibility needs further supplementary data to determine whether the *H*. *miyagawai* mite plays a unique role in urban scrub typhus. Data from the 1990s showed that the northern limit for the presence of *L*. *scutellare* was at the mid-latitude portion of South Korea [28]. Data from the mid-2000s showed that there was northern expansion of *L*. *scutellare* in the western area of South Korea [40]. Finally, the presence of *L*. *scutellare* in a relatively high proportion in Mt. Gwanak suggests that the expansion of its habitat already reached the far northern area of Seoul, in South Korea. An additional national survey could explain the recent overall trend. The mite is identified by specific morphological characteristics. In addition to the phenotypic identification, an 18S rDNA sequencing genotypic method was used. This method could detect 50% and 87.5% of phenotypically identified *H*. *miyagawai* and *L*. *scutellare*, respectively, in our study. This sequencing method would be helpful for less experienced researchers who are not arcarologist to recognize the mite species.

We analyzed subgroup of only twenty-six patients for the detailed clinical features. The cases had mild clinical severity including low APACHE II score, few complication, and no hypokalemia. Although the small number doesn’t represent the whole urban cases, they deserve the first clinical description of urban scrub typhus in Seoul. In this study, we confirmed the presence of the Boryong genotype in the chigger mites collected within Seoul. Further identification of causative strains from multiple areas will help to characterize the overall status. All patients visited the hospital due to fever, and 96.2% of patients had a characteristic maculopapular skin rash. However, 11.5% of patients had no eschar, and the skin rash is often faint and misdiagnosed as a drug reaction following fever medication. In the absence of the characteristic eschar, the initial diagnostic approach may be misleading. If there were a pre-existing epidemiologic knowledge about urban scrub typhus, the differential diagnosis of fever could readily include scrub typhus. In an endemic area with a high percentage of eschar-negative scrub typhus, the proper diagnosis would be much more delayed. We included thirty-five cases (39.8%) of clinical scrub typhus in the analysis. Although these cases were not in the scope of confirmed diagnosis, they were highly suggestive of scrub typhus in terms of clinical and epidemiologic aspects. We also included cases with single cut-off value of ≥ 1:160 IgM antibody. Seroconversion of paired sample is a reasonable diagnostic consensus, and single antibody titer cannot be reliably diagnostic. But single acute phase samples based on locally validated positivity titer criteria also deserves diagnostic usefulness [41, 42]. In South Korea the use of a cutoff value of >1:160 for IgM antibody can differentiate between previous and current infections [19]. We could demonstrate the presence of urban scrub typhus even only with cases of the strict paired seroconversion criteria, but we intended to draw the overall epidemiological situation in Seoul.

In the context of the overall increase in national incidences, our study identified urban scrub typhus using national epidemiologic data. Although the absolute number of annual urban cases was small, there was a steady occurrence of cases. We prospectively confirmed the presence of *O*. *tsutsugamushi* in the causative chigger mites within the city. The major composition of *H*. *miyagawai* mite and its infection with *O*. *tsutsugamushi* are interesting findings. Our study shows the possibility that scrub typhus can spread into a city area under the high incidence pressure of the surrounding area, though the speed of increase and the magnitude of incidence are slow. To predict the possible influx of scrub typhus into a city area, we need to monitor the serial geographical change of the vector distribution and the incidence of the disease. This may be helpful in clinical practices for proper diagnosis and also provide a basis for preventive health policy. The situation in other high burden endemic areas in Asia will be similar. The potential for urban scrub typhus must be considered.

## Supporting Information

S1 Table Reported cases of National Infectious Diseases, 2001–2012. (PDF)

S2 Table Characteristics of the patients, N = 88. (PDF)

S3 Table Identification of mites and *Orientia tsutsugamushi*. (PDF)
