# Supplementary material for: Urbanization of Scrub Typhus Disease in South Korea
Source: PLoS Negl Trop Dis. 2015 May 22;9(5):e0003814. doi: 10.1371/journal.pntd.0003814 (PMC4441427; doi:10.1371/journal.pntd.0003814)
Supplement: S1 Table — (PDF) [file pntd.0003814.s001.pdf]

S1 Table. Reported cases of National Infectious Diseases, 2001-2012

Group		Diseases	No. of reported cases											
			2001	2002	2003	2004	2005	2006	2007	2008	2009	2010	2011	2012
I		Cholera	162	4	1	10	16	5	7	5	0	8	3	0
		Typhoid fever	401	221	199	174	190	200	223	188	168	133	148	129
		Paratyphoid fever	36	413	88	45	31	50	45	44	36	55	56	58
		Shigellosis	927	767	1,117	487	317	389	131	209	180	228	171	90
		Enterohemorrhagic *E. coli*	11	8	52	118	43	37	41	58	62	56	71	58
		Viral hepatitis A	–	–	–	–	–	–	–	–	–	–	5,521	1,197
II		Pertussis	9	21	5	6	11	17	14	9	66	27	97	230
		Tetanus	8	4	8	11	11	10	8	16	17	14	19	17
		Measles	23,060	62	33	11	7	28	194	2	17	114	42	3
		Mumps	1,668	764	1,518	1,744	1,863	2,089	4,557	4,542	6,399	6,094	6,137	7,492
		Rubella	128	24	8	15	12	18	35	30	36	43	53	28
	Viral hepatitis B	Acute	–	–	–	–	–	–	–	–	–	–	462	303
		HBsAg(+) maternity	–	–	–	–	–	–	–	–	–	–	936	2,438
		Perinatal	–	–	–	–	–	–	–	–	–	–	30	26
		Japanese encephalitis	1	6	1	0	6	0	7	6	6	26	3	20
		Varicella	–	–	–	–	1,934	11,027	20,284	22,849	25,197	24,400	36,249	27,763
III		Malaria	2,556	1,799	1,171	864	1,369	2,051	2,227	1,052	1,345	1,772	838	555
		Tuberculosis	34,123	32,010	30,687	31,503	35,269	35,361	34,710	34,157	35,845	36,305	39,557	39,545
		Hansen's disease	79	64	41	43	38	56	12	7	5	6	7	5
		Scarlet fever	49	54	107	80	87	108	146	151	127	106	406	968
		Meningococcal meningitis	11	27	38	8	7	11	4	1	3	12	7	4
		Legionellosis	2	1	3	10	6	20	19	21	24	30	28	25
		Vibrio vulnificus sepsis	41	60	80	57	57	88	59	49	24	73	51	65
		Murine typhus	16	9	9	19	35	73	61	87	29	54	23	41
		Scrub typhus	2,637	1,919	1,415	4,698	6,780	6,480	6,022	6,057	4,995	5,671	5,151	8,604
		Leptospirosis	133	122	119	141	83	119	208	100	62	66	49	28
		Brucellosis	0	1	16	47	158	215	101	58	24	31	19	17
		Rabies	1	1	2	1	0	0	0	0	0	0	0	0
		Hemorrhagic Fever with Renal Syndrome	323	336	392	427	421	422	450	375	334	473	370	364
		AIDS (and HIV infection)	327	397	533	610	680	749	740	797	768	773	888	868
	Syphilis	Primary	–	–	–	–	–	–	–	–	–	–	690	562
		Secondary	–	–	–	–	–	–	–	–	–	–	235	199
		Congenital	–	–	–	–	–	–	–	–	–	–	40	26
		Creutzfeldt-Jacob disease(CJD)	–	–	–	–	–	–	–	–	–	–	29	45
IV		Dengue fever	6	9	14	16	34	35	97	51	59	125	72	149
		Botulism	–	0	3	4	0	1	0	0	1	0	1	0
		Q fever	–	–	–	–	–	6	12	19	14	13	8	10
		West Nile fever	–	–	–	–	–	–	–	–	–	–	0	1
		Emerging infectious disease syndrome	0	0	0	0	0	0	0	0	706,911	56,850	0	0
		Lime Borreliosis	–	–	–	–	–	–	–	–	–	–	2	3
		Melioidosis	–	–	–	–	–	–	–	–	–	–	1	0
		Leishmaniasis	0	1	0	1	0	0	0	0	0	1	–	–
		Babesiosis	0	0	0	0	1	0	0	0	0	0	–	–
		Cryptosporidiosis	0	0	0	0	1	0	0	0	0	0	–	–
		Schistosomiasis	0	0	0	0	0	0	2	1	0	0	–	–

1) Excludes Infectious diseases for Sentinel Surveillance System.

2) No. of notifications contains all case classifications of the disease(Confirmed, Suspected, Asymptomatic carrier) respectively.

3) The Viral hepatitis A, Viral hepatitis B, Syphilis and Creutzfeldt-jacob disease, West Nile fever were newly categorized to mandatory surveillance from sentinel surveillance by revision of "the Infectious Disease Prevention and Control Act" in 2010.

4) The Leishmaniasis, Babesiosis, Cryptosporidiosis and Schistosomiasis were newly categorized to sentinel surveillance from mandatory surveillance by revision of "the Infectious Disease Prevention and Control Act" in 2010.

5) No cases of Diphtheria, Poliomyelitis, Epidemic typhus, Anthrax, Plague, Yellow fever, Viral hemorrhagic fever(Marburg virus, Ebola virus, Lassa virus etc), Smallpox, Severe Acute Respiratory Syndrome(SARS), Avian influenza infection in humans, Novel influenza, Tularemia, Tick-borne Encephalitis, Chikungunya fever were notified during 2001-2012.

6) 0: No notified cases, -: Not notifiable.

7) Emerging infectious disease syndrome of 2009, 2010 is influenza A(H1N1)pdm09.
