# Supplementary material for: Urbanization of Scrub Typhus Disease in South Korea
Source: PLoS Negl Trop Dis. 2015 May 22;9(5):e0003814. doi: 10.1371/journal.pntd.0003814 (PMC4441427; doi:10.1371/journal.pntd.0003814)
Supplement: S2 Table — (PDF) [file pntd.0003814.s002.pdf]

S2 Table. Characteristics of the patients, N=88

Year	Sex	Occupation	Outdoor Activity	Suspected place	Diagnosis, confirmed
2010	M	construction worker	Walking	Mt. Gwanak	Y
2010	F	merchant	gardening	Mt. Gwanak	N
2010	M	construction worker	Walking	Mt. Bukhan	N
2010	F	office worker	gardening	Mt. Gwanak	N
2010	F	housewife	Walking	Mt. Gwanak	Y
2010	F	housewife	harvesting	Mt. Gwanak	N
2010	F	housewife	gardening	Mt. Gwanak	N
2010	F	restaurant worker	gardening	Mt. Gwanak	N
2010	M	construction worker	gardening	Mt. Gwanak	N
2010	F	none	Walking	Mt. Gwanak	Y
2010	F	housewife	leisure (picnic)	city park (Children's grand park)	Y
2010	F	student	leisure (picnic)	Mt. Gwanak	Y
2010	F	teacher	Walking	city park (National Cemetery)	Y
2010	F	office worker	Tennis	city park (Galhyon park)	Y
2010	F	housewife	Walking	Mt. Gwanak	N
2010	F	housewife	gardening	Mt. Daemo	Y
2011	M	driver	Walking	Han river (Jungrangcheon)	Y
2011	F	none	Walking	Mt. Gwanak	Y
2011	F	housewife	Walking	Mt. Yongma	Y
2011	F	housewife	leisure (picnic)	Han river (Haneul park)	N
2011	F	housewife	Walking	Mt. Gwanak	N
2011	M	none	gardening	Mt. Gwanak	Y
2011	F	none	Walking	Mt. Goduk	Y
2011	M	guard	-	-	Y
2011	F	none	gardening	-	Y
2011	F	housewife	Walking	Mt. Wawoo	Y
2012	F	housewife	harvesting	city park (Seoul Olympic park)	Y
2012	F	none	harvesting	city park (Seoul Olympic park)	Y
2012	F	none	-	-	N
2012	M	office worker	harvesting	Mt. Daemo	N
2012	F	housewife	Walking	Mt. Gwanak	Y

2012	F	teacher	leisure (picnic)	Han river	Y
2012	F	office worker	Mowing	Han river	Y
2012	F	housewife	harvesting	-	Y
2012	M	none	Walking	Mt. Bukhan	N
2012	F	housewife	harvesting	Mt. Gwanak	N
2012	F	housewife	harvesting	Mt. Gwanak	N
2012	F	housewife	Walking	Mt. Gwanak	N
2012	F	housewife	gardening	Mt. Gwanak	N
2012	M	student	-	-	Y
2012	M	mechanic	leisure (picnic)	city park (Children's grand park)	Y
2012	F	housewife	harvesting	Mt. Cheonggye	Y
2012	M	none	Walking	Mt. Gwanak.	Y
2012	F	housewife	Walking	Mt. Gwanak	N
2012	M	student	Walking	Mt. Gwanak	Y
2012	F	none	harvesting	city park (Goyeo park)	Y
2012	F	restaurant worker	Walking	Mt. Gwanak	Y
2012	F	private business	Walking	Mt. Gwanak	Y
2012	M	private business	Walking	Mt. Gwanak	Y
2012	F	housewife	gardening	Mt. Gwanak	Y
2012	M	construction worker	Walking	-	Y
2012	M	none	leisure (fishing)	Han river	Y
2012	M	doctor	Walking	Han river	N
2012	F	housewife	leisure (picnic)	Han river (Haneul park)	Y
2012	F	housewife	Walking	city park (Boramae park)	Y
2012	F	restaurant worker	Walking	city park (Boramae park)	N
2012	M	construction worker	Walking	Mt. Gwanak	N
2012	F	housewife	Walking	Mt. Daemo	Y
2012	M	private business	Walking	Mt. Surak	Y
2013	F	housewife	harvesting	Mt. Maebong	N
2013	M	private business	Walking	Mt. Gwanak	N
2013	M	none	gardening	Mt. Gwanak	N
2013	F	private business	Walking	Mt. Gwanak	N
2013	F	housewife	harvesting	Mt. Gwanak	N
2013	F	housewife	harvesting	city park (Hongreung park)	N

2013	F	teacher	Walking	Mt. Cheonggye	Y
2013	M	office worker	Walking	Mt. Gwanak	Y
2013	F	housewife	Walking	Mt. Bukhan	N
2013	M	merchant	Walking	Mt. Gwanak	Y
2013	F	housewife	Mowing	-	Y
2013	F	private business	harvesting	city park (Dalto park)	Y
2013	F	housewife	leisure (picnic)	Mt. Gwanak	Y
2013	F	housewife	harvesting	Mt. Gwanak	Y
2013	F	housewife	Walking	Mt. Gwanak	Y
2013	F	none	harvesting	Mt. Bukhan	Y
2013	F	none	-	city park	Y
2013	F	nurse	gardening	city park (Hangdong park)	Y
2013	M	driver	-	-	N
2013	F	housewife	harvesting	Mt. Gwanak	N
2013	F	housewife	-	-	N
2013	F	housewife	gardening	Mt. Umyon	N
2013	M	office worker	Walking	Mt. Dobong	Y
2013	M	none	Walking	Mt. Umyon	Y
2013	M	none	Walking	Mt. Umyon	Y
2013	M	guard	Walking	Mt. Gwanak	N
2013	F	housewife	Walking	Mt. Gwanak	Y
2013	F	housewife	gardening	Mt. Gwanak	N
2013	M	construction worker	-	-	N

* Harvesting means only trivial collection of fruit or seeds for personal use.
