# Supplementary material for: Urbanization of Scrub Typhus Disease in South Korea
Source: PLoS Negl Trop Dis. 2015 May 22;9(5):e0003814. doi: 10.1371/journal.pntd.0003814 (PMC4441427; doi:10.1371/journal.pntd.0003814)
Supplement: S3 Table — (PDF) [file pntd.0003814.s003.pdf]

S3 Table. Identification of mites and *Orientia tsutsugamushi*

Date collected	ID	Morphological ID	Genetic ID (18S rDNA)*	*O. tsutsugamushi (tsa56)**
Oct.28.2013	H1	*H. miyagawai*	-	+
Oct.28.2013	H2	*H. miyagawai*	+	
Oct.28.2013	H3	*H. miyagawai*	-	
Oct.28.2013	H4	*H. miyagawai*	-	
Oct.28.2013	H5	*H. miyagawai*	+	
Oct.28.2013	H6	*H. miyagawai*	-	
Oct.28.2013	H7	*H. miyagawai*	+	
Oct.28.2013	H8	*H. miyagawai*	-	
Oct.28.2013	H9	*H. miyagawai*	-	
Oct.28.2013	H10	*H. miyagawai*	+	
Oct.28.2013	H11	*H. miyagawai*	-	-
Oct.28.2013	H12	*H. miyagawai*	+	
Oct.28.2013	H13	*H. miyagawai*	+	
Oct.28.2013	H14	*H. miyagawai*	+	
Nov.05.2013	H15	*H. miyagawai*	-	
Nov.05.2013	H16	*H. miyagawai*	-	
Nov.05.2013	H17	*H. miyagawai*	-	
Nov.05.2013	H18	*H. miyagawai*	-	
Nov.05.2013	H19	*H. miyagawai*	-	
Nov.05.2013	H20	*H. miyagawai*	+	
Nov.05.2013	H21	*H. miyagawai*	-	-
Nov.05.2013	H22	*H. miyagawai*	-	
Nov.05.2013	H23	*H. miyagawai*	+	
Nov.05.2013	H24	*H. miyagawai*	+	
Nov.05.2013	H25	*H. miyagawai*	+	
Nov.05.2013	H26	*H. miyagawai*	-	
Nov.05.2013	H27	*H. miyagawai*	+	
Nov.05.2013	H28	*H. miyagawai*	-	
Nov.05.2013	H29	*H. miyagawai*	+	
Nov.05.2013	H30	*H. miyagawai*	+	
Nov.05.2013	H31	*H. miyagawai*	-	-
Nov.05.2013	H32	*H. miyagawai*	-	
Nov.05.2013	H33	*H. miyagawai*	+	
Nov.05.2013	H34	*H. miyagawai*	+	
Nov.05.2013	H35	*H. miyagawai*	-	
Nov.05.2013	H36	*H. miyagawai*	+	
Nov.05.2013	H37	*H. miyagawai*	-	
Nov.05.2013	H38	*H. miyagawai*	-	
Nov.05.2013	H39	*H. miyagawai*	-	
Nov.12.2013	H40	*H. miyagawai*	+	
Nov.12.2013	H41	*H. miyagawai*	+	-
Nov.12.2013	H42	*H. miyagawai*	+	
Nov.12.2013	H43	*H. miyagawai*	-	
Nov.12.2013	H44	*H. miyagawai*	+	
Nov.12.2013	H45	*H. miyagawai*	-	
Nov.19.2013	H46	*H. miyagawai*	+	
Nov.19.2013	H47	*H. miyagawai*	-	
Nov.19.2013	H48	*H. miyagawai*	-	

Nov.19.2013	H49	*H. miyagawai*	-	
Nov.19.2013	H50	*H. miyagawai*	-	
Nov.19.2013	H51	*H. miyagawai*	+	+
Nov.19.2013	H52	*H. miyagawai*	+	
Nov.19.2013	H53	*H. miyagawai*	+	
Nov.19.2013	H54	*H. miyagawai*	+	
Nov.19.2013	H55	*H. miyagawai*	-	
Nov.19.2013	H56	*H. miyagawai*	-	
Nov.19.2013	H57	*H. miyagawai*	-	
Nov.19.2013	H58	*H. miyagawai*	-	
Nov.19.2013	H59	*H. miyagawai*	-	
Nov.19.2013	H60	*H. miyagawai*	-	
Nov.19.2013	H61	*H. miyagawai*	+	_
Nov.19.2013	H62	*H. miyagawai*	+	
Nov.19.2013	H63	*H. miyagawai*	-	
Nov.19.2013	H64	*H. miyagawai*	-	
Nov.19.2013	H65	*H. miyagawai*	-	
Nov.19.2013	H66	*H. miyagawai*	+	
Nov.19.2013	H67	*H. miyagawai*	+	
Nov.19.2013	H68	*H. miyagawai*	+	
Nov.19.2013	H69	*H. miyagawai*	-	
Nov.26.2013	H70	*H. miyagawai*	+	
Nov.26.2013	H71	*H. miyagawai*	+	_
Nov.26.2013	H72	*H. miyagawai*	+	
Nov.26.2013	H73	*H. miyagawai*	+	
Nov.26.2013	H74	*H. miyagawai*	+	
Nov.26.2013	H75	*H. miyagawai*	+	
Nov.26.2013	H76	*H. miyagawai*	+	
Oct.18.2013	L1	*L. scutellare*	+	_
Oct.22.2013	L2	*L. scutellare*	+	
Oct.22.2013	L3	*L. scutellare*	+	
Oct.22.2013	L4	*L. scutellare*	+	
Oct.22.2013	L5	*L. scutellare*	+	
Oct.28.2013	L6	*L. scutellare*	+	
Oct.28.2013	L7	*L. scutellare*	+	
Nov.05.2013	L8	*L. scutellare*	-	
Nov.12.2013	L10	*L. palpale*	-	-
Nov.05.2013	L9	*L. zetum*	-	-
Nov.12.2013	L11	*L. zetum*	-	

*(+), PCR-positive; (-), PCR-negative

ID, identification;
